# Dose and genotype dependent effects of foliar acetic acid on sweet corn under water deficit

**DOI:** 10.1038/s41598-025-26320-6

**Published:** 2025-11-27

**Authors:** Tahoora Batool Zargar, Oqba Basal, Szilvia Veres

**Affiliations:** Faculty of Agricultural and Food Sciences and Environmental Management, Institute of Applied Plant Biology, University of Debrecen, Debrecen, Hungary

**Keywords:** Water deficit, Genetic diversity, Organic acid, Chlorophyll fluorescence, Plant sciences, Plant physiology

## Abstract

Sweet corn (*Zea mays* L. var. saccharata) faces significant challenges due to water deprivation caused by global water scarcity. This study investigates the potential of acetic acid to enhance sweet corn resilience by modulating morphophysiological and biochemical traits under water deficit conditions. Three genotypes were subjected to foliar application of acetic acid at altered concentrations under water deprivation. Water deprivation significantly decreased specific root and shoot length and root volume in all genotypes. However, acetic acid alleviated these adverse effects, particularly in Tyson, which exhibited a notable increase, indicating enhanced adaptability to stress. Specific leaf area increased after treatment with acetic acid; likewise, stomatal conductance showed a significant increase in Messenger and Tyson on application with acetic acid under water deprivation. Chlorophyll-a and chlorophyll-b exhibited genotype-specific and concentration-dependent responses to the treatments, with significant increases observed in Tyson with acetic acid application at 10 mM under water deprivation. Chlorophyll fluorescence parameters varied; while Messenger and GSS 8529 showed non-significant results, Tyson exhibited significant increases in actual photochemical efficiency, mainly with a low concentration of acetic acid under water deprivation. Acetic acid also reduced malondialdehyde levels, a marker of oxidative stress, across all genotypes under stress, and increased peroxidase activity in Tyson and GSS 8529, indicating enhanced antioxidant defences. These findings suggest that acetic acid application effectively mitigates the effects of water deprivation by enhancing photosynthetic efficiency, antioxidant defences, and growth parameters, showing substantial genotype differences. Further research is recommended to optimize acetic acid treatments, considering genotype-specific responses to maximize stress resilience and growth performance.

**Supplementary Information:**

The online version contains supplementary material available at 10.1038/s41598-025-26320-6.

## Introduction

Sweet corn (*Zea mays* L. var. saccharata) is a specific variety of the corn plant that resulted from a mutation in the SU locus (sugary gene) of dent corn, which in the endosperm of the corn kernel regulates the conversion of sugar into starch^1^. This mutation enhances sugar accumulation, resulting in sweet corn having twice as much sugar as regular corn^2^. As the world’s most widely cultivated and economically significant cereal crops it is ranked third in importance to human consumption, after rice and wheat. Farmers increasingly turn to specialty corn production, such as sweet corn, for higher returns and job opportunities, particularly in urban areas. Sweet corn boasts immense market potential and genetic diversity, offering scope for enhancing its nutritional value^3^. Sweet corn has diverse physiological constituents, including sugars, starch, water-soluble polysaccharides, proteins, vitamins, and minerals, contributing to its nutritional value^3,4^.

In addition to being a national favorite in the USA and Canada, sweetcorn is becoming increasingly well-liked in India and other Asian countries. The Iroquois had a significant role in disseminating sweet corn, referred to as Papoon, to European immigrants around 1779 and quickly gained popularity in the southern and central regions of the United States^1^. In contrast to other corn types that are often harvested when mature at the dent stage, sweet corn is selectively chosen at the milking stage, making it more appropriate for use as a vegetable rather than a grain and is highly valuable in the vegetable processing industry (canning and freezing) and is also famous for fresh consumption. However, global water scarcity significantly affects sweet corn’s productivity. Despite their high genetic diversity, maize varieties are only somewhat resistant to drought stress; in particular, the seedling stage of maize is vulnerable to water scarcity^5,6^.

Addressing this issue and improving water use efficiency and drought tolerance in sweet corn is paramount. Drought, after pathogens, is the second major limiting factor for plant productivity, which can restrict 25% of arable land production worldwide^7^. Plants employ various adaptive strategies, including physiological and biochemical alterations, to cope with stressful conditions, ultimately impacting their productivity^8^. Water deficit conditions pose a critical problem for maize growth, affecting anatomy, morpho-physiological, and biochemical processes^9^. Drought-induced osmotic stressors elicit rapid modifications in gene expression and metabolic processes, facilitating plants’ adaptation to demanding environmental circumstances^10,11^. According to Latif et.al.^12^ drought stress in maize significantly impairs root development, resulting in reduced root biomass, volume, and length. In response, the formation of fine roots often increases to enhance water absorption efficiency. Additionally, water deficit conditions lead to a decrease in root surface area and trigger distinct changes in root architecture among maize varieties. The stressors mentioned directly affect the control of stomata, resulting in a decrease in the efficiency of photosynthesis in plants^13^. One of the significant consequences is the adverse impact on the photosynthetic apparatus, often leading to a decrease in chlorophyll content. Additionally, osmotic stresses by water shortages cause the accumulation of reactive oxygen species (ROS), which later are responsible for oxidative reactions like chlorophyll degradation, membrane lipid peroxidation, protein denaturation, and strand breakage of DNA if the imbalance between ROS production and scavenging is not addressed^14^.

Recent studies identified an easily accessible and applicable organic acid, acetic acid (CH_3_COOH), has a good potential for improving plant’s ability to withstand water deprivation by enhancing the rate of photosynthesis, uptake of nutrients, and absorption of water^15–18^. The exogenous application of acetic acid improved tolerance to drought stress in cassava, indicating its potential to ameliorate the effects of drought stress in this crop^19^. Likewise, in *Arabidopsis thaliana*, the enhanced expression of genes associated with acetic acid production improved drought resistance, suggesting that altering the expression of genes related to acetic acid metabolism can improve the capacity of plants to withstand water deficit conditions^20^. According to previous findings, the interaction of acetic acid with phytohormones, such as jasmonic acid, can influence the chromatin structure and the expression of genes, which may affect the transcription of stress-responsive genes, thereby improving the adaptive responses to water stress^15^.

An increase in photosynthesis rates, absorption of water, uptake of nutrients, and interaction with phytohormones to regulate gene expression proposes that acetic acid holds promising potential for agricultural practices aimed at sustainable crop production under unfavorable environmental conditions^15^. By uncovering the role of acetic acid application in drought stress tolerance and exploring its underlying mechanisms, research has opened new possibilities for addressing the challenges posed by water scarcity in agriculture and the environment^15^.

Currently, the planting of sweet corn as seedlings is widespread. However, the protection of sweet corn seedlings under water deficit stress with externally applied acetic acid has not yet been investigated. Therefore, it is hypothesized that the foliar application of acetic acid on different sweet corn genotypes during early vegetative stages under water deprivation will positively affect plant physiology.

## Material and methods

### Plant material, growth conditions, and experimental treatments

The experiment was conducted at the Department of Applied Plant Biology, Institute of Crop Sciences, University of Debrecen, Hungary. It took place in a controlled climate environmental room and focused on three genotypes of sweet corn (*Zea mays* L. var. saccharata): Messenger (provided by Seminis), GSS 8529 (provided by Syngenta), and Tyson (provided by Syngenta). The seeds were washed with tap water multiple times and sterilized by soaking in 6% H_2_O_2_. After 15 min, the seeds were washed with distilled water and soaked in a 0.01 M CaSO_4_ solution for two hours, followed by another thorough wash with distilled water.

The seeds were then germinated geotropically between moistened filter papers at a temperature of 24 °C. The room temperature and humidity were carefully maintained at 24 °C ± 0.2 °C and 62.5% ± 0.5%, respectively. One week after sowing, seedlings (Zadoks scale 10–11)^21^ with good vigor were transplanted into plastic pots (1.7L) under hydroponic conditions. Each genotype had 18 pots (4 plants per pot), with the nutrient solution described by Marschner et al.^22^ being changed every third day.

Two weeks after sowing, when the seedlings had entered the early vegetative phase (Zadoks scale 13–14)^21^, 5% polyethylene glycol (PEG6000, VWR International bvba, Geldenaaksebaan, Leuven, Belgium) was used to osmotically simulate water deprivation for 9 of the 18 pots per genotype. The one-week treatment with PEG induced mild symptoms of water deficiency, which were checked by relative chlorophyll measurements (SPAD values) and stomatal conductance to assess the effectiveness of water deficiency.

Subsequently, on the 25th day after sowing, an acetic acid foliar treatment was applied for five consecutive days using two different concentrations: 20 mM and 10 mM. Pots were arranged following a randomized complete block design with three independent replicates for each treatment. A total of five different treatments were given as follows (+ control): (1) 10 mM CH_2_COOH; (2) 20 mM CH_2_COOH; (3) 5% PEG; (4) 5% PEG + 10 mM CH_2_COOH (5) 5% PEG + 20 mM CH_2_COOH.

**Fig. 1:**
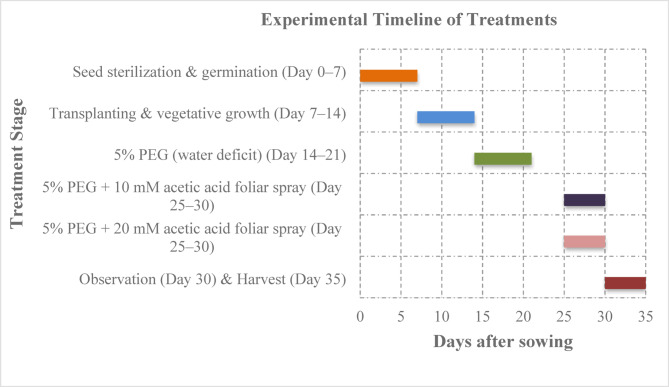
Experimental timeline from seed sterilization to harvest showing treatment progression.

After the 30th day of sowing, relative chlorophyll content (SPAD values), stomatal conductance, and fast phase of the chlorophyll fluorescence induction curve were recorded. Furthermore, the plants were harvested on the 35th day after sowing to analyze various physio-morphological parameters (Fig. 1).

### Plant growth parameters

#### Root and shoot length

The length of the roots and shoots was measured using a ruler. This straightforward method involves physically measuring the distance from the base of the plant to the tip of the root or shoot using a ruler.

#### Root volume

The water displacement technique was used, where the initial and final water levels were measured to calculate the difference, representing the volume of the roots. (Archimedes’ principle)^23^

#### Dry weights of shoot and root

Three replicate samples from each treatment were dried in an oven set at 70 °C for four days to obtain the dry weights. The samples were dried until a consistent weight was achieved and weighed on an electronic scale with a precision of 0.001 g (Ohaus, Switzerland). Specific root (SRL) and shoot (SSL) length, Shoot-to-root ratio (SRR), and Total biomass were calculated.

#### Specific leaf area (SLA)

Five leaf discs with known surface area were used for SLA measurement. The fresh samples were dried at 104.5 °C until a constant weight was gained. The dry weight of all 5 leaf discs was then measured using an electronic scale with a precision of 0.001 g (OHAUS, Swiss). Finally, the SLA of each leaf was calculated by dividing the leaf area by the corresponding leaf dry weight^24^.

#### Relative chlorophyll content (SPAD values)

Relative chlorophyll content was measured using a SPAD-502Plus device (Konica Minolta, Japan). For each treatment, six readings per repetition were recorded from the last fully developed leaves.

#### Photosynthetic pigments

Photosynthetic pigments, including chlorophyll-a and -b and total carotenoid contents, were extracted following the methodology described by Moran and Porath^25^ and determined using the Wellburn,^26^ method. Samples were soaked in darkness in the N, N dimethylformamide solvent for 48 h at room temperature. With the help of UV–VIS spectrophotometry (Metertech SP-830 PLUS, Taiwan), the supernatant was measured after 48 h at specific wavelengths.

#### Xanthophyll pool

To analyze the xanthophyll pool (sum of antheraxanthin, lutein, zeaxanthin, and violaxanthin content), the high-performance liquid chromatography (HPLC) method using a Nucleosil C18 column for separation was used. The eluents contained a mixture of acetonitrile and water in a 9:1 ratio, containing 0.01% triethylamine and ethyl acetate. The HPLC system utilized a UV/VIS detector (JASCO, Japan). Zeaxanthin was regularly injected as a standard compound during the analysis to identify the peaks in the chromatogram and calculate the pigment contents^27^.

#### In-vivo chlorophyll fluorescence parameters

The fast phase of chlorophyll fluorescence induction curve parameters was measured in three replicates from the youngest fully developed leaves with portable chlorophyll fluorometer-PAM-2100 (Walz Gmbh, Germany). Maximum (Fm), minimal (F0), and variable (Fv) fluorescence were recorded to calculate potential photosynthetic capacity (Fv/Fo) and maximum photochemical efficiency of PSII (Fv/Fm). The actual photochemical efficiency of PSII (ΔF/Fm’), also known as Yield, was recorded^28,29^

#### Stomatal conductance

Stomatal conductance was measured using an AP4 porometer (Delta-T, UK) from the youngest fully expanded leaves with three repetitions.

#### Peroxidase (POX) activity

A phosphate buffer was used to homogenize lyophilized leaf samples. After that, the extract was then transferred into an Eppendorf tube and centrifuged for 10 min. In the cuvette, sodium acetate buffer, hydrogen peroxide, and o-anisidine were added to prepare the reaction mixture, and the prepared sample extract was added to the cuvette. Careful mixing of the contents was carried out. Immediately, the absorbance of the reaction mixture was measured at 460 nm using a spectrophotometer (Metertech SP-830 PLUS, Taiwan). Readings were taken every 10 s for a total duration of one minute, enabling to monitor changes in absorbance over time^30^.

#### Malondialdehyde (MDA) Content

Trichloroacetic acid (TCA) was used as an extraction buffer for lyophilized leaf samples. The obtained extract was centrifuged, and the supernatant was transferred to a tube containing TCA and thiobarbituric acid (TBA). At 95°C for 30 min, the mixture was heated to facilitate the reaction, followed by immediate ice cooling to stop further reaction. A second centrifugation step was done to remove any precipitates, and then the absorbance of the resulting solution was measured at 532nm using a spectrophotometer (Metertech SP-830 PLUS, Taiwan)^31^. The absorbance value at this wavelength corresponds to the concentration of the colored complex, indicating the MDA content in the original lyophilized leaf sample.

### Statistical analysis

A randomized complete block design with three replications and four plants per replica was used to arrange the pots. GenStat Release 12.1 was used to perform an analysis of variance (two-way ANOVA) and the Fisher’s protected least significant difference test to analyze the differences between treatments and genotypes to determine statistical significance.

## Results

### Plant growth parameters

#### Specific root and shoot length

The specific root length (SRL) of Messenger, Tyson, and GSS 8529 genotypes was taken as a standard reference in the control group. When sprayed with 10 mM acetic acid, Messenger and GSS 8529 showed reduced SRL (2.78% and 13.9%, respectively), while Tyson substantially increased (71.73%). With 20 mM acetic acid, Messenger and GSS 8529 also experienced decreased SRL (9.43% and 4.47%, respectively), whereas Tyson’s SRL increased (24.88%). When subjected to 5% PEG treatment, all genotypes recorded a significant decrease in SRL (Messenger by 54.13%, Tyson by 56.84%, GSS 8529 by 52.25%). However, combining 10 mM acetic acid with 5% PEG significantly increased SRL for Messenger and Tyson (by 8.1 and 152.5%, respectively, compared to PEG treatment). At the same time, GSS 8529 exhibited a decrease in SRL (by 10.3% compared to PEG treatment). Under 20 mM acetic acid combined with 5% PEG in all genotypes, a significant increase in SRL (Messenger by 50.9%, Tyson by 131.3%, GSS 8529 by 8.4%) compared to PEG treatment was recorded (Table 1).

**Table 1:** Analysis of variance and mean specific root length (cm g^−1^) of three sweet corn genotypes as affected by foliar application of acetic acid under water deprivation.

Variate	Source of variation	d.f	F-value	*p*-value
Specific Root Length	Genotype	2	6321.8	< .001
	Treatment	5	5694.2	< .001
	Genotype. Treatment	10	902.9	< .001

Treatment	Genotypes		
	Messenger	Tyson	GSS 8529
Control	100.57^aA^	80.08^ dB^	75.05^aC^
10 mM acetic acid	97.78^bA^	137.50^aB^	64.62^cC^
20 mM acetic acid	91.12^cA^	99.99^bB^	71.69^bC^
5% PEG	46.11^fA^	34.50^eB^	35.84^eB^
5% PEG + 10 mM acetic acid	49.85^eB^	87.12^cA^	32.15^fC^
5% PEG + 20 mM acetic acid	69.59^ dB^	79.75^dC^	38.85^dA^

*ANOVA results indicate highly significant effects of genotype, treatment, and genotype × treatment interaction (*p* < 0.001). Different lowercase letters denote significant differences among treatments within a genotype; uppercase letters denote significant differences among genotypes within a treatment (*p* ≤ *0.05*) (n = 3).

**Fig. 2:**
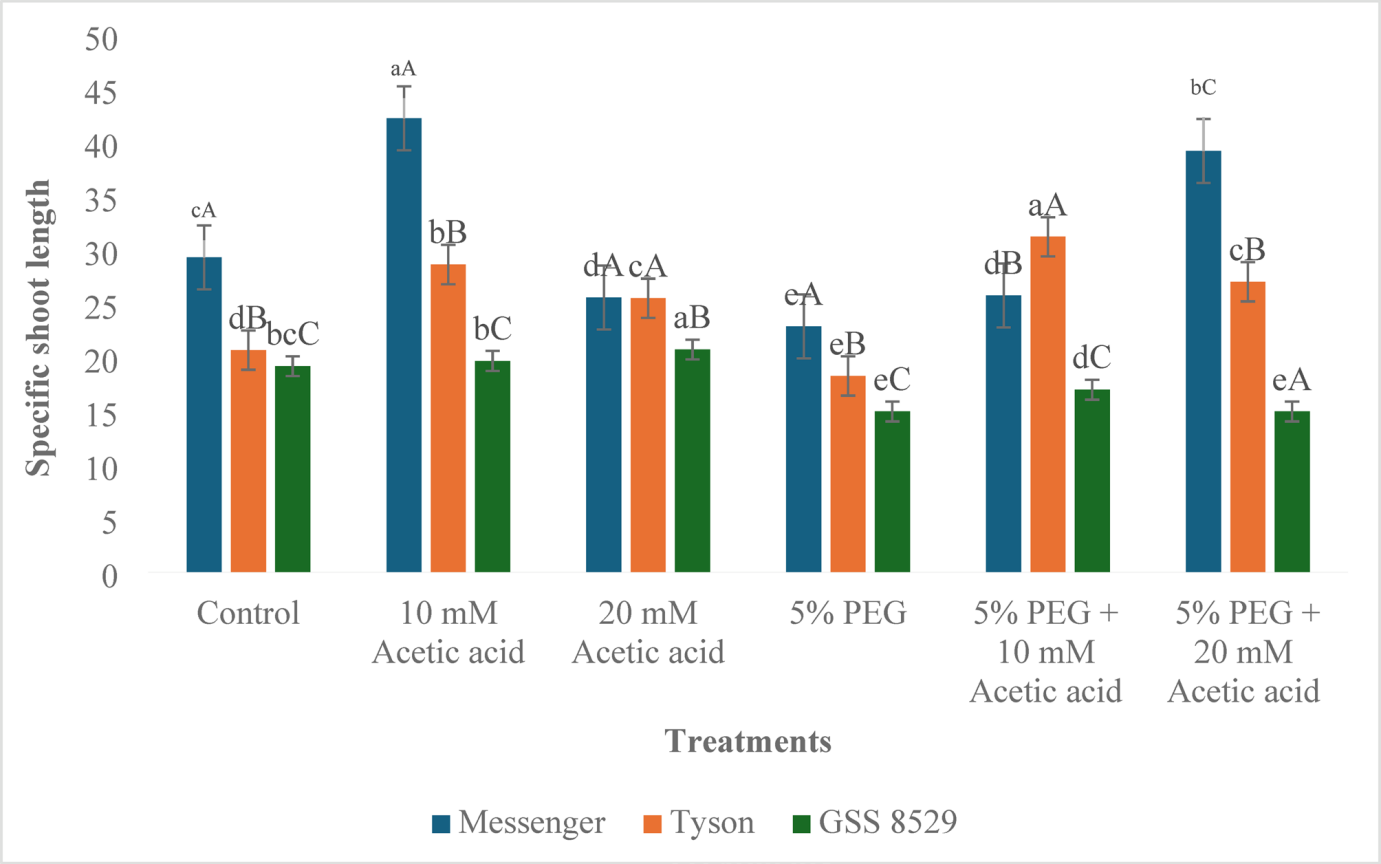
Effect of foliar spray of acetic acid on the specific shoot length (cm g^-1^) of three sweet corn genotypes under water deprivation. Significant differences (*p* ≤ *0.05*) among treatments within genotypes are marked by small letters, and among genotypes within treatments by capital letters (n = 3).

The response of specific shoot length (SSL) differed from SRL; when treated with 10 mM acetic acid, SSL increased in all three genotypes, with significant increases in Messenger and Tyson compared to control (44.1% and 38.6%, respectively). Conversely, 20 mM acetic acid decreased SSL in messenger, but an increase in Tyson and GSS 8529 was recorded. Under PEG treatment, a significant decrease in all three genotypes was recorded (21.9%. 11.6%, and 22.0% in Messenger, Tyson and GSS 8529, respectively). On treatment with 10 mM acetic acid foliar spray under water deprivation, SSL significantly increased in all three genotypes compared to water deficit conditions (12.6%, 70.9%, 13.6%, respectively in Messenger, Tyson, and GSS 8529). Treatment with 20 mM acetic acid recorded no difference in genotype GSS 8529, but an increase of 71.4% and 49.7% were recorded in Messenger and Tyson (Fig. 2, Table 2).

**Table 2:** ANOVA for specific shoot length (cm g^−1^) of three sweet corn genotypes treated with acetic acid under water deprivation.

Variate	Source of variation	d.f	F-value	*p*-value
Specific Shoot Length	Genotype	2	1904.2	< .001
	Treatment	5	402.1	< .001
	Genotype. Treatment	10	174.7	< .001

*ANOVA results indicate highly significant effects of genotype, treatment, and genotype × treatment interaction (*p* < 0.001).

#### Root volume

The root volume (RV) decreased in messenger and GSS 8529 (49.0% and 57.1%) on treatment with 10 mM acetic acid, while in Tyson, a significant increase of 14.3% was recorded. While on treatment with 20 mM acetic acid, a decrease of 7.2% in Tyson and a significant decrease of 60.7% in GSS 8529 was recorded. On treatment with 5% PEG, a significant decrease in all genotypes was recorded (19.6%, 64.3%, and 67.9% in Messenger, Tyson, and GSS 8529, respectively). Treatment with 10 mM acetic acid under PEG increased RV in all three genotypes (34.2%,80.0%,66.7% in Messenger, Tyson, and GSS 8529 respectively) compared to PEG treatment, similarly on treating with 20 mM acetic acid under 5% PEG RV increased in all three genotypes with 299.9% increase in Tyson (Table 3).

**Table 3:** Analysis of variance and mean root volume (cm^3^) of three sweet corn genotypes as affected by foliar application of acetic acid under water deprivation.

Variate	Source of variation	d.f	F-value	*p*-value
Root Volume	Genotype	2	8.6	< .001
	Treatment	5	24.2	< .001
	Genotype. Treatment	10	17.8	< .001

Treatment	Genotypes		
	Messenger	Tyson	GSS 8529
Control	4.33^aB^	4.67^cB^	9.33^aA^
10 mM acetic acid	2.21^aC^	5.33^bA^	4.00^cB^
20 mM acetic acid	4.33^aA^	4.33^cA^	3.67^cdB^
5% PEG	3.48^aA^	1.67^eB^	3.00^dA^
5% PEG + 10 mM acetic acid	4.67^aA^	3.00^dA^	5.00^bA^
5% PEG + 20 mM acetic acid	4.00^aA^	6.67^aB^	3.67^cdA^

*ANOVA results indicate highly significant effects of genotype, treatment, and genotype × treatment interaction (*p* < 0.001). Different lowercase letters denote significant differences among treatments within a genotype; uppercase letters denote significant differences among genotypes within a treatment (*p* ≤ *0.05*) (n = 3).

#### Shoot/root ratio

Shoot: Root (SRR) increased in Messenger and Tyson (9.3% and 64.8%), while decreased in GSS 8529 (8.6%) on treatment with 10 mM acetic acid compared to control. At higher concentrations of acetic acid in Messenger and Tyson, an increase in shoot: root was recorded, while in GSS 8529, a decrease was recorded compared to the control. Upon treatment with 5% PEG, a significant decrease in two genotypes (45.6% and 23.2% in Messenger and GSS 8529, respectively) was recorded, while in Tyson, an increase was recorded. On treatment with 10 mM acetic acid under PEG, a decrease in all three genotypes was recorded (15.0%, 28.9%, and 8.2% in Messenger, Tyson, and GSS 8529, respectively). Likewise, a higher concentration of acetic acid under PEG decreased shoot: root compared to PEG (17.3%, 19.9%, 12.4% in Messenger, Tyson, and GSS 8529, respectively) (Table 4).

**Table 4:** Analysis of variance and mean shoot-to-root ratio (g g^-1^) of three sweet corn genotypes as affected by foliar application of acetic acid under water deprivation.

Variate	Source of variation	d.f	F-value	*p*-value
Shoot: Root	Genotype	2	34.9	< .001
	Treatment	5	24.1	< .001
	Genotype. Treatment	10	4.0	< .001

Treatment	Genotypes		
	Messenger	Tyson	GSS 8529
Control	4.75^aB^	3.43^cdC^	6.29^aA^
10 mM acetic acid	5.19^aA^	5.63^aA^	5.75^abA^
20 mM acetic acid	4.93^aB^	4.77^abB^	6.09^abA^
5% PEG	2.58^bB^	4.51^bcA^	4.83^bcA^
5% PEG + 10 mM acetic acid	2.20^bC^	3.20^ dB^	4.44^cA^
5% PEG + 20 mM acetic acid	2.14^bA^	3.61^cdB^	4.23^cB^

*ANOVA results indicate highly significant effects of genotype, treatment, and genotype × treatment interaction (*p* < 0.001). Different lowercase letters denote significant differences among treatments within a genotype; uppercase letters denote significant differences among genotypes within a treatment (*p* ≤ *0.05*) (n = 3).

#### Total biomass

Treatment with acetic acid decreased total biomass in all three genotypes in both concentrations of acetic acid compared to control (Table 5). However, under 5% PEG treatment, an increase of 10.5% was recorded in GSS 8529. In contrast, a decrease was recorded in Messenger and Tyson, and treatment with 10 mM acetic acid under PEG increased total biomass in Messenger by 41.9% and in GSS 8529 by 18.2%. While on application with 20 mM acetic acid, a decrease in total biomass was recorded in all genotypes compared to PEG treatment.

**Table 5:** Analysis of variance and mean total biomass (g) of three sweet corn genotypes as affected by foliar application of acetic acid under water deprivation.

Variate	Source of variation	d.f	F-value	*p*-value
Dry Biomass	Genotype	2	259.1	< .001
	Treatment	5	15.8	< .001
	Genotype. Treatment	10	6.1	< .001

Treatment	Genotypes		
	Messenger	Tyson	GSS 8529
Control	2.98^cA^	3.52^dA^	4.77^abB^
10 mM acetic acid	1.75^aA^	2.43^aA^	4.22^aB^
20 mM acetic acid	2.72^bcA^	3.00^cA^	4.10^aB^
5% PEG	2.17^abA^	2.94^bcA^	5.27^bB^
5% PEG + 10 mM acetic acid	3.08^cA^	2.75^abcA^	6.23^cB^
5% PEG + 20 mM acetic acid	1.88^aA^	2.53^abA^	4.65^abB^

*ANOVA results indicate highly significant effects of genotype, treatment, and genotype × treatment interaction (*p* < 0.001). Different lowercase letters denote significant differences among treatments within a genotype; uppercase letters denote significant differences among genotypes within a treatment (*p* ≤ 0.05) (n = 3).

#### Specific leaf area

**Fig. 3:**
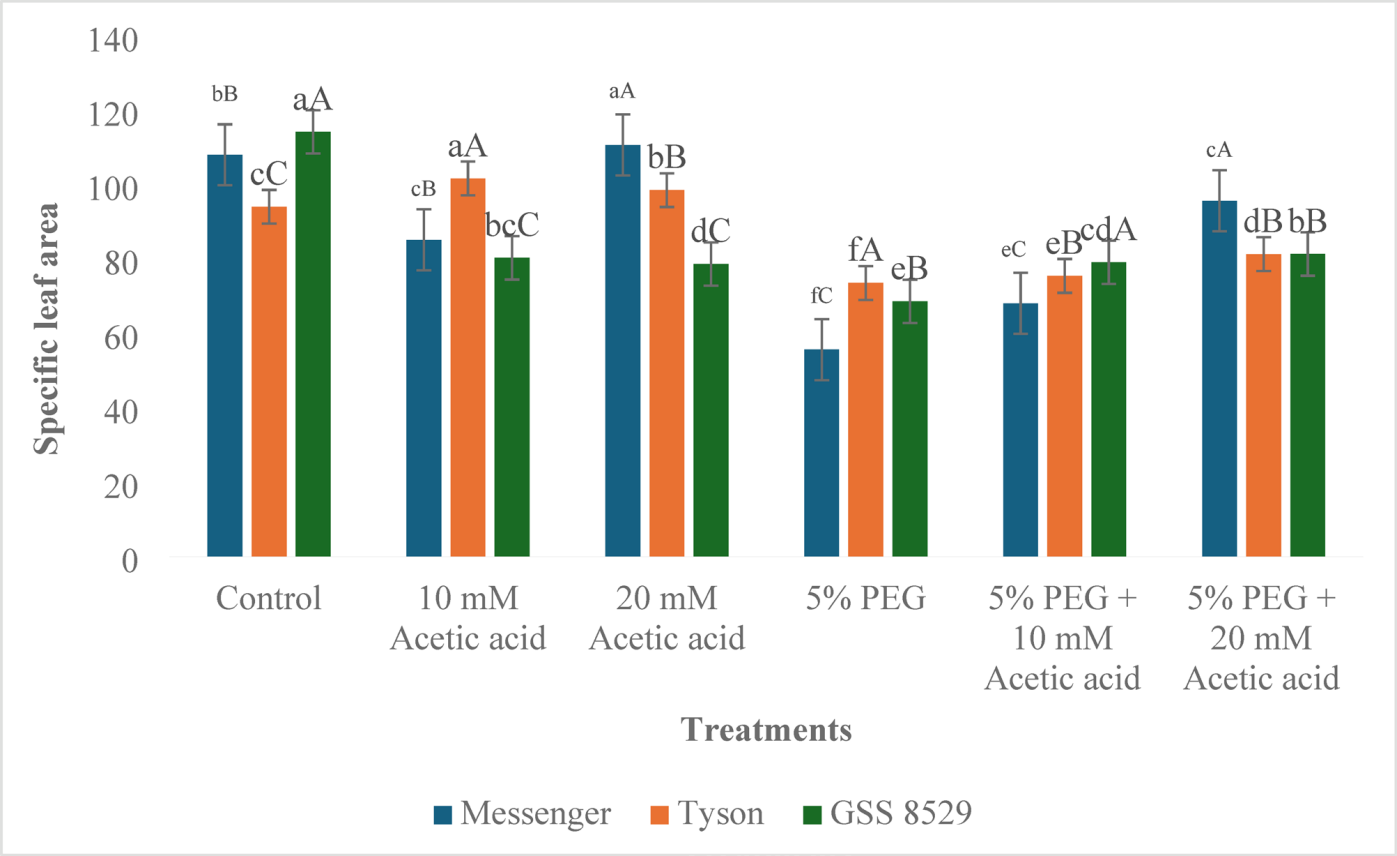
Effect of foliar spray of acetic acid on specific leaf area (cm^2^ g^-1^) of three sweet corn genotypes under water deprivation. Significant differences (*p* ≤ *0.05*) among treatments within genotypes are marked by small letters, and among genotypes within treatments by capital letters (n = 3).

Specific leaf area (SLA) decreased in Messenger and GSS 8529 on treatment with 10 mM acetic acid concentration, while in Tyson, an increase was recorded compared to control. An increase in 20 mM acetic acid application in Messenger and Tyson was recorded, while in GSS 8529, a decrease of 31.2% was recorded in comparison to the control (Fig. 3). Under 5% PEG treatment, SLA decreased in all three genotypes (48.5%, 21.8%, 40.0% in Messenger, Tyson, and GSS 8529 respectively). In combination, treatment of 10 mM acetic acid and 5% PEG increase in all three genotypes were recorded (22.4%, 2.6%, 15.3% in Messenger, Tyson, and GSS 8529 respectively) compared to PEG treatment. In 20 mM acetic acid treatment under PEG, an increase was also recorded compared to PEG treatment (72.0%, 10.6%, and 18.6% in Messenger, Tyson and GSS 8529, respectively) (Table 6).

**Table 6:** ANOVA for specific leaf area (cm^2^ g^-1^) of three sweet corn genotypes treated with acetic acid under water deprivation.

Variate	Source of variation	d.f	F-value	*p*-value
Specific Leaf Area	Genotype	2	172.0	< .001
	Treatment	5	4043.6	< .001
	Genotype. Treatment	10	857.3	< .001

*ANOVA results indicate highly significant effects of genotype, treatment, and genotype × treatment interaction (*p* < 0.001).

### Physiological and biochemical parameters

#### Relative chlorophyll content

Relative chlorophyll content (SPAD) decreased in all three genotypes under PEG treatment (15.4%, 22.4%, and 6.3% in Messenger, Tyson, and GSS 8529, respectively) compared to control. However, under combination treatment of acetic acid and PEG, an increase in SPAD was recorded in all three genotypes compared to PEG treatment (Table 7). On application with 10 mM acetic acid under PEG, a 24.5%, 32.7%, and 24.2% increase were recorded in Messenger, Tyson and GSS 8529. Under PEG treatment, 20 mM acetic acid application increased SPAD by 28.3%, 38.3%, and 21.9%, respectively.

**Table 7:** Analysis of variance and mean relative chlorophyll content (SPAD values) of three sweet corn genotypes as affected by foliar application of acetic acid under water deprivation.

Variate	Source of variation	d.f	F-value	*p*-value
SPAD	Genotype	2	309.1	< .001
	Treatment	5	545.6	< .001
	Genotype. Treatment	10	42.5	< .001

Treatment	Genotypes		
	Messenger	Tyson	GSS 8529
Control	27.52^bA^	27.65^dA^	27.80^cA^
10 mM acetic acid	31.07^eB^	26.91^dC^	33.68^aA^
20 mM acetic acid	29.97^dC^	32.77^aB^	33.68^aA^
5% PEG	23.27^aB^	21.47^eC^	26.05^dA^
5% PEG + 10 mM acetic acid	28.97^cB^	28.50^cB^	32.35^bA^
5% PEG + 20 mM acetic acid	29.85^ dB^	29.67^bB^	31.75^bA^

*ANOVA results indicate highly significant effects of genotype, treatment, and genotype × treatment interaction (*p* < 0.001). Different lowercase letters denote significant differences among treatments within a genotype; uppercase letters denote significant differences among genotypes within a treatment (*p* ≤ 0.05) (n = 6).

### Photosynthetic pigments

#### Chlorophyll-a content

The chlorophyll-a content (Chl-a) exhibited a distinct response to different treatments in each genotype. In Messenger, a decrease in Chl-a content was recorded in all treatments, with a significant decrease in acetic acid concentrations compared to the control. However, no significance was recorded in PEG treatment, nor was the application of acetic acid under PEG treatment. In Tyson, a decrease in acetic acid and PEG treatment was recorded (21.8%, 21.5%, 38.8% in 10 mM acetic acid, 20 mM acetic acid, and 5% PEG treatment, respectively) compared to control. However, a 68.6% and 46.4% increase was recorded in 10 mM acetic acid and 20 mM acetic acid application under PEG treatment compared to PEG treatment alone. While in GSS 8529, an increase in both acetic acid concentrations and PEG treatment was recorded compared to the control (Table 8).

**Table 8:** Analysis of variance and mean photosynthetic pigments (mg g^−1^) of three sweet corn genotypes as affected by foliar application of acetic acid under water deprivation.

Variate	Source of variation	d.f	F-value	*p*-value
Chlorophyll- a	Genotype	2	7.7	0.0
	Treatment	5	5.2	0.0
	Genotype. Treatment	10	9.9	< .001
Chlorophyll- b	Genotype	2	4.6	0.0
	Treatment	5	8.0	< .001
	Genotype. Treatment	10	8.2	< .001
Total Carotenoids	Genotype	2	0.8	0.4
	Treatment	5	3.9	0.0
	Genotype. Treatment	10	5.9	< .001

Trait	Treatment	Messenger	Tyson	GSS 8529
Chlorophyll- a	Control	13.69^aA^	12.95^aB^	9.83^aC^
	10 mM Acetic acid	12.17^bA^	10.13^cB^	11.81^aA^
	20 mM acetic acid	10.14^cB^	10.16^cB^	12.49^aA^
	5% PEG	12.50^bA^	7.93^ dB^	12.38^aA^
	5% PEG + 10 mM acetic acid	12.00^bA^	13.37^aA^	12.19^aA^
	5% PEG + 20 mM acetic acid	12.47^bA^	11.61^bA^	11.75^aA^
Chlorophyll- b	Control	5.89^aA^	6.48^aA^	2.93^aB^
	10 mM Acetic acid	5.19^abA^	3.32^cB^	3.60^aB^
	20 mM acetic acid	3.03^cB^	3.156^cB^	4.05^aA^
	5% PEG	4.29^bcA^	1.91^ dB^	3.98^aA^
	5% PEG + 10 mM acetic acid	3.82^bcA^	4.71^bA^	3.95^aA^
	5% PEG + 20 mM acetic acid	4.45^abcA^	4.10^bcA^	4.29^aA^
Total carotenoids	Control	3.89^aA^	2.09^cB^	2.07^aB^
	10 mM Acetic acid	2.39^bA^	2.13^cA^	2.41^aA^
	20 mM acetic acid	1.92^bB^	2.29^bcA^	2.54^aA^
	5% PEG	2.62^bA^	3.25^aA^	2.78^aA^
	5% PEG + 10 mM acetic acid	2.50^bA^	2.93^abA^	2.64^aA^
	5% PEG + 20 mM acetic acid	2.59^bA^	2.49^bcA^	2.58^aA^

*ANOVA results indicate highly significant effects of genotype, treatment, and genotype × treatment interaction (*p* < 0.001). Different lowercase letters denote significant differences among treatments within a genotype; uppercase letters denote significant differences among genotypes within a treatment (*p* ≤ 0.05) (n = 3).

#### Chlorophyll-b content

The chlorophyll-b content (Chl-b) also showed varied responses to the different treatments in each genotype (Table 9). Under 10 mM acetic acid treatment, Messenger and Tyson experienced a decrease (11.9% and 48.8%, respectively), while GSS 8529 showed an increase (22.9%). When treated with 20 mM acetic acid, Messenger and Tyson showed a significant decrease (48.57% and 51.2%), while in GSS 8529, a significant increase of 38.2% was recorded. Under the 5% PEG treatment, the Chl-b content in Messenger and Tyson decreased significantly (27.2% and 70.5%), and in GSS 8529, an increase (35.8%) was recorded. When treated with a combination of 10 mM acetic acid and 5% PEG, Tyson showed a significant increase of 146.6% compared to PEG treatment (Table 8). On treatment with a combination of 20 mM acetic acid and 5% PEG, all three genotypes showed an increase in Chl-b content: Messenger by 3.7%, Tyson by 114.7%, and GSS 8529 by 7.8% compared to PEG treatment.

**Table 9:** ANOVA for xanthophyll content (µg µL^-1^) of three sweet corn genotypes treated with acetic acid under water deprivation.

Variate	Source of variation	d.f	F-value	*p*-value
Xanthophyll content	Genotype	2	40.0	< .001
	Treatment	5	3.4	0.0
	Genotype. Treatment	10	3.2	0.0

*ANOVA results indicate highly significant effects of genotype, treatment, and genotype × treatment interaction (*p* < 0.001).

#### Total carotenoids

The total carotenoid content in genotype Messenger recorded a decrease in both acetic acid concentrations and PEG treatments compared to control (38.6%, 50.6%, 32.2%, respectively); a further decrease was recorded in combination treatments of acetic acid and PEG in comparison to PEG treatment. In Tyson, an increase of 1.9%, 9.6%, and 55.5% was recorded in 10 mM, 20 mM acetic acid and PEG treatment, respectively, compared to control, and a decrease was recorded in combination treatment compared to PEG treatment. In GSS 8529, an increase was recorded in acetic acid concentrations and PEG treatment compared to control (16.4%, 22,7%, and 34.3%, respectively). In contrast, a further decrease was recorded compared to PEG treatment under a combination treatment of PEG and acetic acid (Table 8).

#### Xanthophyll content

**Fig. 4:**
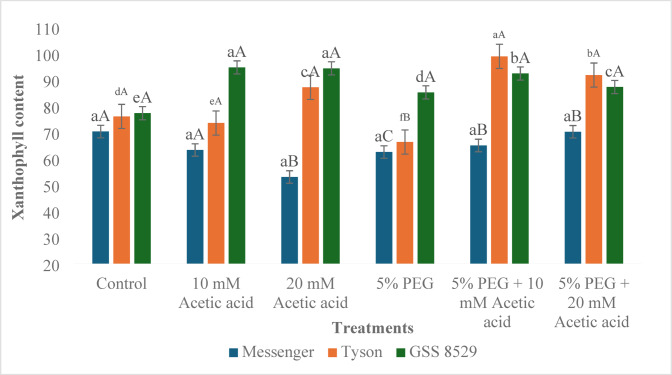
Effect of foliar spray of acetic acid on xanthophyll content (µg µL^−1^) of three sweet corn genotypes under water deprivation. Significant differences (*p* ≤ 0.05) among treatments within genotypes are marked by lowercase letters, and among genotypes within treatments by uppercase letters (n = 3).

Xanthophyll content in Messenger decreased in acetic acid treatments and 5% PEG treatment (10.0%, 24.6%, 11.1% respectively) in comparison to control, while under combination treatment of acetic acid and PEG increase (4.2%, 12.3% in 10 mM and 20 mM acetic acid) was recorded compared to PEG treatment. In Tyson, xanthophyll content decreased in 10 mM acetic acid and PEG treatment (3.3% and 12.8%, respectively). In combination with the treatment of acetic acid and PEG, a significant increase (49.2%, 38.5% in 10 mM, and 20 mM) was recorded compared to PEG treatment. In genotype GSS 8529, an increase in xanthophyll content was recorded in acetic acid treatments and PEG treatments compared to control and in combination treatment with acetic acid and PEG (8.4%, 2.4% in in10 mM and 20 mM, respectively) in comparison to PEG treatment (Fig. 4).

#### Actual photochemical efficiency of photosystem II

The actual photochemical efficiency of photosystem II is also known as yield (ΔF/Fm’). In Messenger and GSS 8529, non-significant results were recorded for all treatments. However, in Tyson under 10 mM acetic acid treatment, a significant increase of 6.2% and a 5.1% increase in 20 mM acetic acid treatment was recorded compared to the control. In combination treatment of 10 mM acetic acid and PEG treatment, a significant increase of 3.1% was recorded compared to PEG treatment (Table 10).

**Table 10:** Analysis of variance and mean ΔF/Fm’, Fv/Fo, Fv/Fm of three sweet corn genotypes as affected by foliar application of acetic acid under water deprivation.

Variate	Source of variation	d.f	F-value	*p*-value
Fv/Fo	Genotype	2	2.8	0.1
	Treatment	5	10.4	< .001
	Genotype. Treatment	10	1.1	0.4
Fv/Fm	Genotype	2	0.2	0.9
	Treatment	5	3.7	0.0
	Genotype. Treatment	10	1.3	0.3
ΔF/Fm’	Genotype	2	0.3	0.7
	Treatments	5	3.9	0.0
	Genotype. Treatments	10	2.1	0.0

Trait	Treatment	Messenger	Tyson	GSS 8529
ΔF/Fm’	Control	0.73^aAB^	0.71^cB^	0.75^aA^
	10 mM Acetic acid	0.74^aA^	0.75^abA^	0.74^aA^
	20 mM acetic acid	0.74^aA^	0.75^abA^	0.74^aA^
	5% PEG	0.74^aA^	0.73^bA^	0.73^aA^
	5% PEG + 10 mM acetic acid	0.75^aA^	0.76^aA^	0.75^aA^
	5% PEG + 20 mM acetic acid	0.74^aA^	0.75^abA^	0.73^aA^
Fv/F0	Control	3.38^bA^	3.00^aA^	3.16^cA^
	10 mM Acetic acid	3.93^aA^	3.58^aB^	3.44^bcB^
	20 mM acetic acid	3.46^bA^	3.23^aA^	3.72^abA^
	5% PEG	4.13^aA^	4.04^aA^	3.73^abB^
	5% PEG + 10 mM acetic acid	3.86^aA^	3.90^aA^	3.93^aA^
	5% PEG + 20 mM acetic acid	4.11^aA^	3.75^aA^	4.02^aA^
Fv/Fm	Control	0.77^bA^	0.74^aA^	0.76^aA^
	10 mM Acetic acid	0.80^aA^	0.75^aA^	0.77^abA^
	20 mM acetic acid	0.78^bA^	0.76^aA^	0.79^bcA^
	5% PEG	0.81^aA^	0.80^aA^	0.79^bcB^
	5% PEG + 10 mM acetic acid	0.79^aA^	0.80^aA^	0.80^cA^
	5% PEG + 20 mM acetic acid	0.80^aA^	0.89^aA^	0.80^cA^

*ANOVA results indicate highly significant effects of genotype, treatment, and genotype × treatment interaction (*p* < 0.001). Different lowercase letters denote significant differences among treatments within a genotype; uppercase letters denote significant differences among genotypes within a treatment (*p* ≤ 0.05) (n = 3) and for yield (n = 12).

#### Potential photosynthetic capacity

Potential Photosynthetic Capacity (Fv/Fo) under 10 mM acetic acid foliar spray led to a considerable increase in Fv/F0, with Messenger showing a rise of 16.3% and Tyson demonstrating a 19.3% increase compared to the control. Additionally, Tyson exhibited an increase of 34.7% with the application of 5% PEG. However, when combined with 10 mM acetic acid, Tyson’s increase dropped to 30%, indicating a decrease compared to the PEG treatment alone. Conversely, in GSS 8529, an increase with the combined treatments was recorded, mainly with 20 mM acetic acid and 5% PEG (7.7%) compared to the PEG treatment.

#### Maximum photochemical efficiency

In the Messenger genotype, increases were recorded in maximum photochemical efficiency (Fv/Fm) with 10 mM acetic acid (3.3%), PEG (4.4%) in comparison to control, but combining 10 mM acetic acid with PEG decreased by 1.3% was recorded compared to PEG treatment. Genotype Tyson showed minor changes, except for 20 mM acetic acid (2.7%) and PEG (8.1%) compared to the control. GSS 8529 genotype exhibited increases with all treatments (10 mM acetic acid—2.2%, 20 mM acetic acid—3.9%, PEG—4.0%), compared to control and with slight enhancements in combined treatments compared to PEG treatment (1.1% and 1.5% in 10 mM and 20 mM acetic acid respectively).

#### Stomatal conductance

Stomatal conductance on applying 10 mM acetic acid decreased in Messenger and Tyson, while in GSS 8529, an increase of 10.6% was recorded. Likewise, with the application of 20 mM, acetic acid decreased stomatal conductance in Tyson and GSS 8529, and an increase of 86.3% was recorded in Messenger. Under PEG treatment, in all three genotypes, a decrease in stomatal conductance was recorded (67.2%, 85.3%, and 28.9% in Messenger, Tyson, and GSS 8529, respectively). In combination treatment with 10 mM acetic caid and PEG, an increase of 321.4% and 265.8% in Messenger and Tyson compared to PEG treatment. Under 20 mM acetic acid and PEG treatment, an increase of 105% and 96.1% were recorded in Messenger and Tyson, while in GSS 8529, a decrease was recorded compared to PEG treatment (Table 11).

**Table 11:** Analysis of variance and mean stomatal conductance (mmol H_2_O m^−2^ s^-1^) of three sweet corn genotypes as affected by foliar application of acetic acid under water deprivation.

Variate	Source of variation	d.f	F-value	*p*-value
Stomatal Conductance	Genotype	2	251.6	< .001
	Treatment	5	396.8	< .001
	Genotype. Treatment	10	279.5	< .001

Treatment	Genotypes		
	Messenger	Tyson	GSS 8529
Control	18.80^cB^	32.80^aA^	15.33^aC^
10 mM acetic acid	7.77^eC^	11.03^cB^	16.96^aA^
20 mM acetic acid	35.03^aA^	9.80^ dB^	7.98^cB^
5% PEG	6.17^fB^	4.83^eC^	10.90^bA^
5% PEG + 10 mM acetic acid	26.00^bA^	17.67^bB^	10.33^bC^
5% PEG + 20 mM acetic acid	12.70^dA^	9.47^ dB^	5.80^dC^

*ANOVA results indicate highly significant effects of genotype, treatment, and genotype × treatment interaction (*p* < 0.001). Different lowercase letters denote significant differences among treatments within a genotype; uppercase letters denote significant differences among genotypes within a treatment (*p* ≤ 0.05) (n = 3).

#### Peroxidase activity

Peroxidase activity (POX) increased on treatment with both acetic acid concentrations in Tyson and GSS 8529, while a decrease was recorded in Messenger. Under 5% PEG treatment increase in all three genotypes was recorded (18.1%, 51.0%, and 13.5% in Messenger, Tyson and GSS 8529, respectively). On application with 10 mM acetic acid under 5% PEG peroxidase activity decreased in Messenger and Tyson (19.1% and 39.1%, respectively), while GSS 8529 increase was recorded compared to PEG treatment. While on application with 20 mM acetic acid under 5%PEG, a decrease in Messenger and an increase in Tyson and GSS 8529 (20.9% and 91.7%) was recorded compared to PEG treatment (Table 12).

**Table 12:** Analysis of variance and mean peroxidase activity (µmol min^- 1^ g^-1^) of three sweet corn genotypes as affected by foliar application of acetic acid under water deprivation.

Variate	Source of variation	d.f	F-value	*p*-value
Peroxidase activity	Genotype	2	1390.7	< .001
	Treatment	5	177.4	< .001
	Genotype. Treatment	10	68.6	< .001

Treatment	Genotypes		
	Messenger	Tyson	GSS 8529
Control	50.56^cA^	20.69^dC^	23.78^ dB^
10 mM acetic acid	44.69^eA^	31.56^bC^	34.76^bB^
20 mM acetic acid	44.61^eA^	29.35^cC^	33.37^bB^
5% PEG	59.71^aA^	31.25^bcB^	26.98^cC^
5% PEG + 10 mM acetic acid	48.30^dA^	19.03^dC^	32.90^bB^
5% PEG + 20 mM acetic acid	54.76^bA^	37.78^aB^	51.72^aA^

*ANOVA results indicate highly significant effects of genotype, treatment, and genotype × treatment interaction (*p* < 0.001). Different lowercase letters denote significant differences among treatments within a genotype; uppercase letters denote significant differences among genotypes within a treatment (*p* ≤ 0.05) (n = 3).

#### Malondialdehyde content

**Fig. 5:**
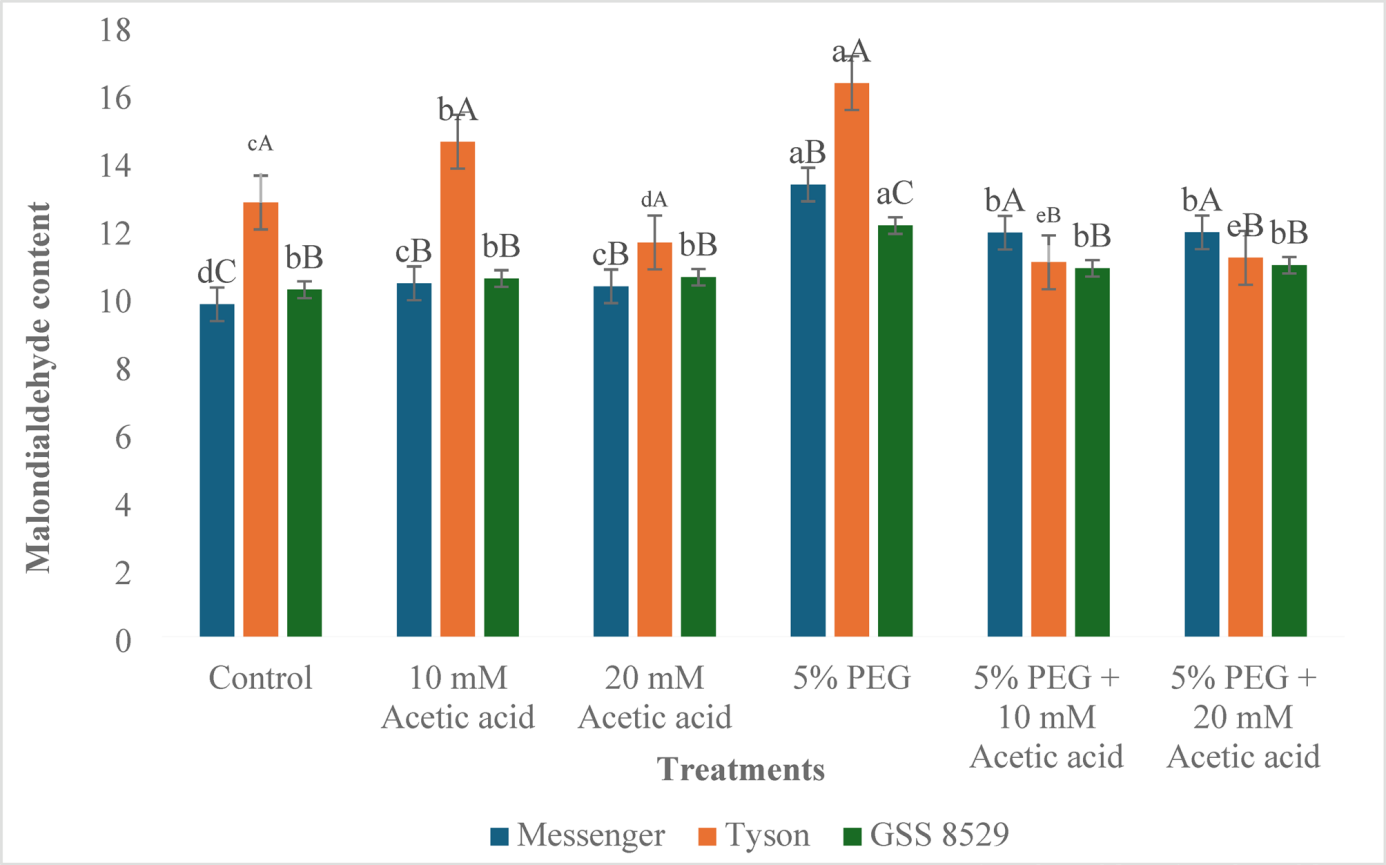
Effect of foliar spray of acetic acid on malondialdehyde content (nmol g^-1^) of three sweet corn genotypes under water deprivation. Significant differences (*p* ≤ 0.05) among treatments within genotypes are marked by lowercase letters, and among genotypes within treatments by uppercase letters (n = 3).

Malondialdehyde content (MDA) often indicates plant oxidative stress. A higher MDA content suggests more elevated levels of stress. MDA increased in all three genotypes, Messenger, Tyson, and GSS 8529, by 6.3%, 14.0%, and 3.2%, respectively, compared to the control under 10 mM acetic acid foliar spray. 20 mM acetic acid increased MDA in Messenger and GSS 8529 by 5.4% and 3.6%, respectively, but decreased in Tyson by 9.2% compared to control. 5% PEG increased the MDA content in all three genotypes, Messenger, Tyson, and GSS 8529, by 36.1%, 27.5% and 18.5%, respectively, compared to control. Combining 10 mM acetic acid with 5% PEG reduced MDA by 10.7%, 32.4%, and 10.4%, respectively, in Messenger, Tyson, and GSS 8529 compared to PEG. 20 mM acetic acid with 5% PEG reduced MDA by 10.6%, 31.6%, and 9.7% compared to the 5% PEG treatment in Messenger, Tyson, and GSS 8529 (Fig. 5) (Table 13).

**Table 13:** ANOVA for malondialdehyde content (nmol g^-1^) of three sweet corn genotypes treated with acetic acid under water deprivation.

Variate	Source of variation	d.f	F-value	*p*-value
MDA	Genotype	2	232.1	< .001
	Treatment	5	130.7	< .001
	Genotype. Treatment	10	46.2	< .001

*ANOVA results indicate highly significant effects of genotype, treatment, and genotype × treatment interaction (*p* < 0.001).

## Discussion

The primary objective of this study was to evaluate the physiological responses of three different sweet corn genotypes to foliar acetic acid application, including Messenger, Tyson, and GSS 8529, under water deficit conditions. The study aimed to comprehend the possible alleviating impact of acetic acid on plant development, morphology, and stress resilience.

The impact of drought stress on global agricultural productivity is a substantial concern, which calls for developing novel strategies to improve plant resilience. Drought significantly affects plant physiology and growth, resulting in stomatal closure, decreased rate of photosynthesis, and disturbed leaf growth^32–34^. The application of acetic acid, a well-known regulator of various physiological processes in plants exogenously, has been proposed as a potential solution to mitigate the adverse impacts of osmotic stress, particularly in drought and salinity^15,19,35^. Moreover, studying the relationship between acetic acid and PEG, a commonly used method to induce water deprivation, presents an intriguing opportunity to study the potential synergistic or antagonistic effects on plant responses^14^. In this experiment, Messenger, Tyson, and GSS 8529 sweetcorn genotypes were selected for their distinct characteristics and potential to respond differently to environmental stress factors. A thorough evaluation of how different genotypes respond to acetic acid and PEG-induced water deprivation, precise measurements of several morphological, physiological, and biochemical traits, including specific root and shoot length, root volume, total biomass, stomatal conductance photosynthetic pigments, antioxidant enzymatic activity, and chlorophyll fluorescence was done.

The analysis of results recorded for specific root and shoot length demonstrate that different genotypes exhibit diverse reactions to varying concentrations of acetic acid, both when applied individually and in conjunction with water deprivation. Growth parameter reduction is one of the apparent outcomes of water deprivation in plants. In this study, PEG-mediated water deprivation was manifested as the reduction in specific shoot and root length in all observed genotypes, which aligns with the finding that in the rapid vegetative stage, the water stress restricts plant growth^36,37^. For example, Messenger, Tyson, and GSS 8529 showed decreases in SRL of 54.1%, 56.8%, and 52.2%, respectively, compared with control (Table 1). Meristematic cell division and cell elongation contribute to plant growth, and water deficit in meristematic cells severely affects cell elongation, reducing cell division and, ultimately, plant growth^36^. Osmotic stress disrupts water equilibrium, affecting plant cells’ turgor, growth, and division, decreasing shoot length and dry shoot weight^38^. Exogenous application of acetic acid in higher concentrations under normal conditions is generally toxic; however, under water deficit conditions, application at this concentration proved beneficial in several plant species, including maize^15,18^. In genotype Messenger and GSS 8529, our results align with this study as individual treatments of acetic acid decreased the SRL, while application of both acetic acid concentrations under water deprivation increased the SRL in both genotypes with a prominent increase at higher concentration (Messenger by 50.9%, GSS 8529 by 8.4% compared to PEG treatment) (Table 1). Meanwhile, in Tyson, a significant increase was recorded in individual acetic acid treatments and under water deprivation. This could be possible because of different physiological responses to acetic acid and different genetic compositions of Tyson, enabling tolerance or gaining advantages from the used concentrations of acetic acid. Similar to SRL, in SSL on treatment with acetic acid, improvement was recorded compared to water deficit conditions, suggesting that water deficit induced reduction in plant growth indices, but the application of acetic acid mitigated the adverse effects (Fig. 2). Our results aligned with Sun et al.^39^ that acetic acid treatment reduced impact of drought stress on fresh weight and above-ground plant height by maintaining photosynthetic rate and decreasing the breakdown of pigments.

Drought stress commonly induces modifications in root system architecture that help plants adapt by enhancing water uptake capacity and maintaining osmotic balance. Under water deficit, plants often exhibit increased root: shoot ratios, deeper rooting, and changes in specific root length, which reflect adaptive osmoregulatory mechanisms^40,41^. Root elongation under stress facilitates access to deeper soil moisture, while finer roots with higher SRL improve soil exploration efficiency^42^. At the cellular level, drought triggers osmotic adjustment through accumulation of compatible solutes such as proline, glycine betaine, and soluble sugars, which help maintain cell turgor and sustain root growth^32,43^. In our study, PEG induced water deprivation reduced root volume in all genotypes, indicating impaired root expansion, yet acetic acid alleviated this reduction, suggesting a possible role in stabilizing osmotic adjustment. Similar improvements in root growth under exogenous acetic acid application have been attributed to enhanced proline accumulation and antioxidant defense, which collectively support osmoregulation under drought [14.15]. Thus, the genotype-specific changes in SRL and root volume observed here may reflect differential capacity for osmotic adjustment and root system plasticity in response to combined PEG and acetic acid treatments.

The interrelationship between osmoregulation and root attributes is particularly relevant in explaining the genotype-specific responses observed in this study. Osmotic adjustment, primarily through the accumulation of compatible solutes such as proline, total free amino acids, soluble sugars, and water-soluble proteins, enables root cells to maintain turgor pressure and water uptake under water deficit, thereby supporting continued elongation and fine root development^44–46^. These osmolytes also play a protective role in maintaining enzyme activity and stabilizing membranes under water scarcity. The enhancement of enzymatic antioxidants such as peroxidase, catalase, and glutathione peroxidase further contributes to ROS detoxification, thereby maintaining cellular homeostasis and sustaining metabolic function during drought^47,48^. This mechanism is consistent with the observed improvements in specific root length and root volume under combined PEG and acetic acid treatments, particularly in Tyson, where enhanced root proliferation suggests effective coordination between osmotic balance, antioxidant defense, and root plasticity. Conversely, the reduced root growth in Messenger and GSS 8529 under stress conditions highlights a weaker osmoregulatory capacity, which limits their ability to sustain root expansion. At the whole-plant level, root system modifications such as changes in SRR and SRL can alleviate drought effects by improving soil exploration and water acquisition, thus reducing osmotic stress in aerial tissues^49–51^. The beneficial effects of acetic acid observed in this study may therefore be attributed to its ability to enhance osmolyte accumulation and antioxidant activity, thereby stabilizing osmotic adjustment and facilitating root growth under PEG-induced water deprivation^14,15,17^. These findings collectively suggest that drought tolerance in sweet corn is not solely dependent on osmotic adjustment at the cellular level but also on the extent to which root system traits can translate this adjustment into sustained water uptake capacity, as also indicated by previous studies in maize and Arabidopsis^14,18^.

Root volume was significantly reduced in all genotypes, which is consistent with the anticipated effects of water deprivation and highlights the root growth susceptibility to changes in water supply. Water deprivation-induced reduction in root growth might be attributed to a decrease in specific root length (Table 1), ultimately reducing root volume^52^. An acetic acid application under water deprivation alleviates the adverse effects on RV, suggesting that acetic acid may help mitigate the deleterious consequences of water deprivation induced by PEG. On application with 10 mM acetic under water deprivation, a higher increase in RV compared to water deficit conditions was recorded in Messenger (34%) and GSS 8529 (67%). In contrast, Tyson 20 mM acetic acid (300%) showed more favorable results, suggesting concentration and genotype-specific response towards acetic acid treatment (Table 3). Our results align with Mahmud et al.^14^, found that treating maize and Arabidopsis with acetic acid under PEG-induced osmotic stress showed similar RV as in control plants, suggesting the alleviating nature of acetic acid to overcome adverse effects of osmotic stress.

The shoot-to-root ratio results revealed that water deprivation decreased SRR in Messenger and GSS 8529, which coincided with the results for the above growth traits. The results in these genotypes demonstrate that the SRR based on dry weight decreased in the presence of water deprivation, indicating that shoot growth is more sensitive than root growth, as also described by Ashraf and Foolad^53^. In Tyson, SRR increased under water deprivation, indicating genotype-specific response, as reported that different genotypes of durum wheat responded differently under PEG-induced water deficit^54^. Several studies^17,19,35^ have reported that both PEG-induced osmotic stress and water deficiency can lead to the suppression of shoot and root growth, along with a reduction in various growth parameters. Individual treatment, especially with lower concentration of acetic acid, increased SRR in Messenger and Tyson. In contrast, in combination treatment with water deprivation, a decrease was reported compared to water deficit conditions, indicating the possibility of the beneficial effects of acetic acid or detrimental interactions between acetic acid and water deprivation (Table 4). These results indicate that acetic acid treatment can affect plant growth differently depending on concentration, genotype, and combination with water deprivation.

Total dry biomass decreased in two genotypes, Messenger and Tyson when exposed to water deprivation, while in GSS 8529, an increase in biomass was recorded in the same condition. These findings suggest that response to water deprivation can vary among genotypes based on genetic or physiological differences^32,55^. The water deficit can limit various physiological processes, leading to decreased biomass production^56^. Individual treatment with both concentrations of acetic acid used decreased total dry biomass in all genotypes. At the same time, a lower concentration of acetic acid under water deprivation increased biomass in Messenger (42%) and GSS 8529 (18%) while higher concentration reduction was recorded. These concentration-specific responses highlight the intricate dynamics between acetic acid and PEG treatments, parallel to previous research suggesting acetic acid toxicity in general but can maintain plant viability in situations with a shortage of water^18^. Our results agree with Kudo et al.^18^, who described foliar acetate toxicity under non-stress, but contrast under water deficit where acetate acted beneficially. This dual role emphasizes the need to contextualize acetic acid application.

Osmotic stress reduces the surface leaf area by reducing leaf expansion as water deficit restricts cell division and expansion, resulting in smaller and narrower leaves with reduced leaf area^57^. Our results align with this study, as SLA was reduced in all genotypes under PEG-induced water deprivation. The lack of moisture leads to a decrease in both the number and size of leaves. Leaf growth is often influenced by turgor pressure and the availability of assimilates. Under dry environmental conditions, the primary factors that restrict leaf growth are decreased turgor pressure and a reduced rate of photosynthesis^58^. Foliar application of acetic acid under water deprivation increased the SLA in all genotypes with a pronounced increase under 20 mM acetic acid application. Aligning with our results, in mung bean plants under salt stress, an increase in leaf area per trifoliate was recorded on foliar application of acetic acid^17^. Along with the decrease in SLA, relative chlorophyll content also decreased under PEG-induced water deprivation, indicating a reduction in chlorophyll content. This suggests water deprivation negatively affects chlorophyll production in all three genotypes^59^. On application of acetic acid under water deprivation, SPAD values increased in all genotypes, suggesting a potential protective role of acetic acid against water deprivation on chlorophyll content at both concentrations with 10 mM acetic acid under PEG, a 24.5%, 32.7%, and 24.2% increase were recorded in Messenger, Tyson and GSS 8529. Under PEG treatment, 20 mM acetic acid application increased SPAD by 28.3%, 38.3%, and 21.9%, respectively (Table 7).

Chlorophyll-a and chlorophyll-b content decreased in Messenger and Tyson under individual acetic acid treatments and water deprivation, while in GSS 8529, an increase in Chl-a and Chl-b was recorded. Drought may have a negative effect on photosynthetic pigments and thylakoid membranes, resulting in decreased chlorophyll levels^60,61^. Nevertheless, there are conflicting results since several studies suggest a rise in chlorophyll levels in cereals when subjected to moisture stress, emphasizing the impact of crop species and variety^62^. For instance, multiple cultivars of *Vigna mungo* L. hepper (black gram) showed varying levels of chlorophyll content under water stress conditions. The varied response across each cultivar in chlorophyll concentrations can be attributed to the variations in the activity of enzymes involved in chlorophyll production^63^.

Under drought stress, higher chlorophyll-a content was recorded than chlorophyll-b content in some cases^64^, while in Brassica species, the ratio of chlorophyll-a to chlorophyll-b was decreased^65^, suggesting the complex connection between drought stress, the production of chlorophyll and the varied responses shown by various crops and cultivars. In Tyson, the application of acetic acid under water deprivation increased Chl-a and Chl-b content; a lower concentration of acetic acid has more pronounced effects than a higher one, suggesting a mitigative role of acetic acid under water deprivation. According to Rahman et al.^35^, acetic acid treatment in soybean under drought stress maintains photosynthetic pigments by either synthesizing photosynthetic pigments or slowing down the breakdown. Similar results were recorded in mung beans under salinity^17,66^.

Total carotenoid content results suggest the intricate relationship between acetic acid concentrations, water deprivation, and genotype-specific responses. Under water deprivation, total carotenoid content increased in Tyson and GSS 8529, indicating the protective response towards stress^67^, while in Messenger, the reduction was recorded, suggesting a possible decline as of reduced photosynthetic activity or chlorophyll degradation^68^. In Tyson and GSS 8529, total carotenoid content increased compared to control under acetic acid treatments, suggesting alleviating capacity of acetic acid to overcome water deprivation.

Xanthophyll accumulation usually increases under water deprivation, but the severity and the duration of stress, as well as genetic variations and physiological responses of cultivars, can change the magnitude of xanthophyll accumulation under water deprivation^69,70^. In our study, xanthophyll accumulation decreased in Messenger and Tyson under PEG-induced water deprivation, while in GSS 8529, an increase was recorded. On treatment with acetic acid under water deprivation, an increase in xanthophyll accumulation at both concentrations was recorded, suggesting a protective response towards water deprivation^67^.

The variable chlorophyll fluorescence parameters, potential photosynthetic capacity (Fv/Fo), maximum photochemical efficiency of photosystem II (Fv/Fm) and actual photochemical efficiency of PSII (ΔF/Fm’), also known as yield, are crucial for assessing the health of a leaf internal apparatus during photosynthesis and detecting and quantifying plant tolerance to drought stress^70–72^. Studies have shown that these parameters strongly correlate with whole-plant mortality in response to environmental stresses and are reliable stress indicators^70,72,73^. Chlorophyll fluorescence parameters (Fv/Fo, Fv/Fm, ΔF/Fm′) did not show major treatment effects, remaining stable between ~ 0.74–0.81 across genotypes (Table 10). This indicates that acetic acid influenced pigment pools and oxidative balance rather than altering intrinsic PSII efficiency. In Tyson, an increase in yield was recorded on application with 10 mM acetic acid under water deprivation compared to water deficit conditions. The results suggest that the response of these physiological variables to environmental stresses may vary depending on the specific genotype being studied. Tyson recorded an increase in yield on application with 10 mM acetic acid under water deprivation, highlighting the potential benefits of this treatment in certain genotypes.

Stomatal conductance recorded a varied response in acetic acid treatments in all studied genotypes. Underwater deprivation, a significant reduction in stomatal conductance suggests a decrease in water loss by stomatal closure. This is reflected as a first response under moisture stress and reduces carbon dioxide intake, leading to poor photosynthesis^32^. Applying acetic acid in Messenger and Tyson recorded an improvement in stomatal conductance, while in GSS 8529, a decrease was recorded under water deprivation. Reduced stomatal conductance on treatment with acetic was also recorded in previous research in mung beans and cotton plants^17,74^.

Peroxidase is a crucial antioxidant enzyme that helps manage oxidative stress by neutralizing reactive oxygen species. The differences in peroxidase activity suggest that different concentrations of acetic acid, water deprivation, genetic variations, and physiological responses of different genotypes have varied effects on antioxidant enzymes. All three genotypes under water deficit conditions recorded an increase in peroxidase activity, aligning with previous studies^75,76^. Treatment with acetic acid under water deprivation increased POX activity in GSS 8529 with both concentrations, while in Tyson, only a higher concentration led to an increase in POX activity. In Messenger, POX decreased on treatment with acetic acid under water deprivation. A decrease in POX was also recorded in soyabean on treatment with acetic acid^35^, suggesting species and genotype-specific responses. Peroxidase (POX) activity increased under PEG in all genotypes, consistent with oxidative stress signaling^75,76^. Acetic acid further elevated POX in Tyson and GSS 8529 but decreased it in Messenger (Table 12). At 10 mM acetic acid without PEG, POX and MDA both increased in Tyson, suggesting that acetic acid alone induced a transient oxidative burst, activating defense responses rather than alleviation.

Malondialdehyde, a byproduct of lipid peroxidation, is widely known as a reliable marker for oxidative stress in plants^77^. Under PEG-induced water deprivation, MDA accumulation increased in all genotypes (Messenger, Tyson, and GSS 8529, by 36.1%, 27.5% and 18.5%),

indicating the rise of reactive oxygen species under stress and oxidative damage. Treatment with acetic acid resulted in a decrease in MDA accumulation, resulting in a reduction of reactive oxygen species and subsequently reduced MDA accumulation. These results align with the previous study where acetic acid in soyabean reduced the MDA levels under drought stress by an increase in the activity of antioxidant enzymes^35^. These reductions are not fully explained by POX activity but likely reflect broader priming of ROS-scavenging and pigment stabilization, consistent with Kim et al.^15^, Mahmud et al.^16^, and Kudo et al.^18^.

## Conclusion

This study assessed the morpho-physiological and biochemical responses of Messenger, Tyson, and GSS 8529 sweet corn genotypes to foliar acetic acid application under water deprivation. However, the growth metrics were adversely affected by water deprivation, but acetic acid treatment under water deprivation showed both genotype-specific hazards and ameliorative responses. Acetic acid alone often reduced biomass and increased MDA, confirming its hazardous role under non-stress, but under PEG induced water deprivation, acetic acid reduced MDA, improved pigments, and in some cases recovered biomass. Based on our criteria, “hazardous” treatments were those decreasing biomass and/or increasing MDA compared with control (e.g., Messenger and GSS 8529 under acetic acid alone), whereas “non-toxic/beneficial” treatments were those increasing pigments or biomass and/or reducing MDA under water deprivation (e.g., Tyson under both concentrations, Messenger and GSS 8529 under 10 mM acetic acid + PEG).

These findings demonstrate that acetic acid functions as a context-dependent regulator of stress physiology. While hazardous under normal growth, it can act as a protective priming agent under water deprivation, stabilizing pigments, reducing oxidative damage, and recovering biomass. Importantly, genotype-specific responses highlight the need for tailoring concentration and treatment strategies for each genotype. Further investigation is required to study mechanistic basis for these differences.

## Supplementary Information

Below is the link to the electronic supplementary material.

Supplementary Material 1

## Data Availability

The data generated and analyzed during this study is included in the article.

## Declarations

### Plant material

The collection of plant material complies with relevant institutional, national, and international guidelines and legislation.
