# Supplementary material for: Dose and genotype dependent effects of foliar acetic acid on sweet corn under water deficit
Source: Sci Rep. 2025 Nov 27;15:42327. doi: 10.1038/s41598-025-26320-6 (PMC12660718; doi:10.1038/s41598-025-26320-6)
Supplement: Supplementary file 1 — Supplementary Material 1 [file 41598_2025_26320_MOESM1_ESM.docx]

| Variate | Source of variation | d.f. | MS | F-value | p-value |  |
| --- | --- | --- | --- | --- | --- | --- |
| Specific Root Length | Genotype | 2 | 5259.2 | 6321.8 | <.001 |  |
|  | Treatment | 5 | 4737.1 | 5694.2 | <.001 |  |
|  | Genotype. Treatment | 10 | 751.2 | 902.9 | <.001 |  |
|  | Residual | 36 | 0.8 |  |  |  |
|  | Total | 53 |  |  |  |  |
| Specific Shoot Length | Genotype | 2 | 701.4 | 1904.2 | <.001 |  |
|  | Treatment | 5 | 148.1 | 402.1 | <.001 |  |
|  | Genotype. Treatment | 10 | 64.3 | 174.7 | <.001 |  |
|  | Residual | 36 | 0.4 |  |  |  |
|  | Total | 53 |  |  |  |  |
| Root Volume | Genotype | 2 | 4.0 | 8.6 | <.001 |  |
|  | Treatment | 5 | 11.3 | 24.2 | <.001 |  |
|  | Genotype. Treatment | 10 | 8.3 | 17.8 | <.001 |  |
|  | Residual | 36 | 0.5 |  |  |  |
|  | Total | 53 |  |  |  |  |
| Shoot : Root | Genotype | 2 | 12.5 | 34.9 | <.001 |  |
|  | Treatment | 5 | 8.6 | 24.1 | <.001 |  |
|  | Genotype. Treatment | 10 | 1.4 | 4.0 | <.001 |  |
|  | Residual | 36 | 0.4 |  |  |  |
|  | Total | 53 |  |  |  |  |
| Dry Biomass | Genotype | 2 | 30.5 | 259.1 | <.001 |  |
|  | Treatment | 5 | 1.9 | 15.8 | <.001 |  |
|  | Genotype. Treatment | 10 | 0.7 | 6.1 | <.001 |  |
|  | Residual | 36 | 0.1 |  |  |  |
|  | Total | 53 |  |  |  |  |
| Specific Leaf Area | Genotype | 2 | 78.7 | 172.0 | <.001 |  |
|  | Treatment | 5 | 1849.7 | 4043.6 | <.001 |  |
|  | Genotype. Treatment | 10 | 392.1 | 857.3 | <.001 |  |
|  | Residual | 36 | 0.5 |  |  |  |
|  | Total | 53 |  |  |  |  |
| Chl.a | Genotype | 2 | 5.9 | 7.7 | 0.0 |  |
|  | Treatment | 5 | 4.0 | 5.2 | 0.0 |  |
|  | Genotype. Treatment | 10 | 7.7 | 9.9 | <.001 |  |
|  | Residual | 36 | 0.8 |  |  |  |
|  | Total | 53 |  |  |  |  |
| Chl.b | Genotype | 2 | 2.1 | 4.6 | 0.0 |  |
|  | Treatment | 5 | 3.6 | 8.0 | <.001 |  |
|  | Genotype. Treatment | 10 | 3.7 | 8.2 | <.001 |  |
|  | Residual | 36 | 0.5 |  |  |  |
|  | Total | 53 |  |  |  |  |
| Fv/Fo | Genotype | 2 | 0.2 | 2.8 | 0.1 |  |
|  | Treatment | 5 | 0.9 | 10.4 | <.001 |  |
|  | Genotype. Treatment | 10 | 0.1 | 1.1 | 0.4 |  |
|  | Residual | 36 | 0.1 |  |  |  |
|  | Total | 53 |  |  |  |  |
| Fv/Fm | Genotype | 2 | 0.0 | 0.2 | 0.9 |  |
|  | Treatment | 5 | 0.0 | 3.7 | 0.0 |  |
|  | Genotype. Treatment | 10 | 0.0 | 1.3 | 0.3 |  |
|  | Residual | 36 | 0.0 |  |  |  |
|  | Total | 53 |  |  |  |  |
| MDA | Genotype | 2 | 21.0 | 232.1 | <.001 |  |
|  | Treatment | 5 | 11.8 | 130.7 | <.001 |  |
|  | Genotype. Treatment | 10 | 4.2 | 46.2 | <.001 |  |
|  | Residual | 36 | 0.1 |  |  |  |
|  | Total | 53 |  |  |  |  |
| Peroxidase | Genotype | 2 | 2387.6 | 1390.7 | <.001 |  |
|  | Treatment | 5 | 304.6 | 177.4 | <.001 |  |
|  | Genotype. Treatment | 10 | 117.8 | 68.6 | <.001 |  |
|  | Residual | 36 | 1.7 |  |  |  |
|  | Total | 53 |  |  |  |  |
| Stomatal Conductance | Genotype | 2 | 191.9 | 251.6 | <.001 |  |
|  | Treatment | 5 | 302.8 | 396.8 | <.001 |  |
|  | Genotype. Treatment | 10 | 213.3 | 279.5 | <.001 |  |
|  | Residual | 36 | 0.8 |  |  |  |
|  | Total | 53 |  |  |  |  |
| Xanthophylls | Genotype | 2 | 2916.3 | 40.0 | <.001 |  |
|  | Treatment | 5 | 247.8 | 3.4 | 0.0 |  |
|  | Genotype. Treatment | 10 | 233.3 | 3.2 | 0.0 |  |
|  | Residual | 36 | 73.0 |  |  |  |
|  | Total | 53 |  |  |  |  |
| Total Carotenoids | Genotype | 2 | 0.1 | 0.8 | 0.4 |  |
|  | Treatment | 5 | 0.5 | 3.9 | 0.0 |  |
|  | Genotype. Treatment | 10 | 0.8 | 5.9 | <.001 |  |
|  | Residual | 36 | 0.1 |  |  |  |
|  | Total | 53 |  |  |  |  |
| SPAD | Genotype | 2 | 94.1 | 309.1 | <.001 |  |
|  | Treatment | 5 | 166.1 | 545.6 | <.001 |  |
|  | Genotype. Treatment | 10 | 12.9 | 42.5 | <.001 |  |
|  | Residual | 90 | 0.3 |  |  |  |
|  | Total | 107 |  |  |  |  |
| Yield | Genotype | 2 | 0.0 | 0.3 | 0.7 |  |
|  | Treatments | 5 | 0.0 | 3.9 | 0.0 |  |
|  | Genotype. Treatments | 10 | 0.0 | 2.1 | 0.0 |  |
|  | Residual | 198 | 0.0 |  |  |  |
|  | Total | 215 |  |  |  |  |
